# Susceptibility of clinical *Candida* species isolates to antifungal agents by E-test, Southern Iran: A five year study

**Published:** 2011-12

**Authors:** P Badiee, A Alborzi

**Affiliations:** Prof. Alborzi Microbiology Research Center, Mycology Department, Shiraz University of Medical Sciences, Shiraz, Iran

**Keywords:** *Candida*, amphotericin B, itraconazole, voriconazole, antifungal susceptibility, E-test

## Abstract

**Background and Objectives:**

The incidence of fungal infections in immunocompromised patients, especially by *Candida* species, has increased in recent years. This study was designed to identify *Candida* species and determine antifungal susceptibility patterns of 595 yeast strains isolated from various clinical specimens.

**Material and Methods:**

Identification of the isolates were determined by the API 20 C AUX kit and antifungal susceptibilities of the species to fluconazole, amphotericin B, ketoconazole, itraconazole, voriconazole, and caspofungin were determined by the agar-based E-test method.

**Results:**

*Candida albicans* (48%) was the most frequently isolated species, followed by *Candida kruzei* (16.1%), *Candida glabrata* (13.5%), *Candida kefyr* (7.4%), *Candida parapsilosis* (4.8%), *Candida tropicalis* (1.7%) and other species (8.5%). Resistance varies depending on the species and the respective antifungal agents. Comparing the MIC90 for all the strains, the lower MIC90 was observed for caspofungin (0.5 µg/ml). The MIC90 for all *Candida* species were 64 µg/ml for fluconazole, 0.75 µg/ml for amphotericin B, 4 µg/ml for ketoconazole, 4 µg/ml for itraconazole, and 2 µg/ml for voriconazole.

**Conclusions:**

Species definition and determination of antifungal susceptibility patterns are advised for the proper management and treatment of patients at risk for systemic candidiasis. Resistance to antifungal agents is an alarming sign for the emerging common nosocomial fungal infections.

## INTRODUCTION

Systemic candidiasis (SC) is the most common invasive fungal infection as the nosocomial infection in patients undergoing major surgeries during prolonged neutropenia, transplantation and extended hospital stays of days to weeks, (1). This infection is potentially a life-threatening complication in immunocompromised patients. The introduction of novel antineoplastic agents, antifungals, antibacterial and antivirals over the past 10 years has led to a shift in fungal epidemiology (2, 3) and fever without specific signs and symptoms of localized fungal infection is the most common clinical presentation. Intensive and long-term use of antifungals leads to a decline in sensitivity and resistance development of *Candida* strains (4). Antifungal resistance surveillance serves as a major strategy for prophylaxis, empirical therapy, and treatment of SC. For the management of patients suffering from SC, determination of the changes in the distribution of *Candida* species and respective sensitivity pattern to antifungal agents are important.

Antifungal prophylaxis is warranted in patients with developing risk of SC. As definitive early diagnosis is difficult, empiric therapy of antifungal agents has become a standard of practice in immune-suppressed patients like neutropenic patients who had received broad spectrum antibacterial therapy but remain persistently febrile. The antifungal susceptibility testing of pathogenic fungi can manage the selection of adequate therapy and also provide an estimate of antifungal efficacy. Monitoring of drug resistance development can predict therapeutic outcome and therapeutic potential of untested compounds (5–7).

The purpose of this study was to determine the distribution of *Candida* species and *in vitro* susceptibilities of antifungal agents against the *Candida* isolated from the patients referred to a mycology center in southern Iran using E-test for the best management and treatment of those at risk for SC.

## MATERIALS AND METHODS

This study was designed in mycology department, Clinical Microbiology Research Center, Shiraz University of Medical Sciences, southern Iran from October 2005 to October 2010. Clinical samples including mouth, blood, abdominal tap, urine, sputum, esophageal, oropharyngeal, vagina, biopsy and broncho alveolar lavage of patients were cultured on saboaroud dextrose agar (Merck, Germany) and incubated at 24°C for 10 days. All *Candida spp*. isolated were cultured on potato dextrose agar (OXOID LTD, Basingstoke, Hampshire, England) twice for 48h at 35°C for the purity inspected. For species typing of the isolates, germ tube and chlamydospore production tests were performed. The carbohydrate assimilation patterns of all the isolates were studied using the API 20 C AUX system according to the manufacturer's procedure (Biomerieux, France). *Candida* (C) *dubliniensis* sp. was recognized by molecular assay from *Candida albicans* (8) because these species have the same pattern for carbohydrate assimilation. *Candida parapsilosis* ATCC 22019 and *Candida krusei* ATCC 6258 (9) were quality controls and tested each day for all antifungal agents.

Susceptibility test for the isolates was performed by the agar-based E-test method (Biomeriux, Sweden) with RPMI 1640, 8.4 gram per litter (RPMI; Sigma Chemical Co., St. Louis, Mo.), 2% glucose and1.5% agar which buffered to PH 7.0 with 0.165 M morpholinepropanesulfonic acid buffer (Sigma, St. Louis, Mo.), poured in 90-mm-diameter plates. The plates were inoculated by dipping a sterile swab into the inoculum suspension adjusted to the turbidity of a 0.5 McFarland standard (106 cells/ml) and streaking it across the surface of the agar in four directions. The plates were dried at ambient temperature for 15 minutes before applying the E-test strips. The minimum inhibition concentrations (MIC) endpoints were determined after 24 and 48 h of incubation at 35°C. The MIC was read for amphotericin B, as the drug concentration that zone determined the point of complete inhibition (100%), and for ketoconazole; itraconazole, voriconazole and caspofungin the significant inhibition decreased 80% of the growth. The resistance breakpoints for antifungals are amphotericin B > 1.0; fluconazole ≥ 64; itraconazole ≥ 1.0; voriconazole ≥ 8.0; ketoconazole ≥ 4.0; and caspofungin ≥ 2.0 micrograms per milliliter (10–15). MIC50 and MIC90 (the MIC at which 50% and 90% of the isolates are inhibited) were also calculated.

Data were entered into SPSS version 16 and were subsequently analyzed using descriptive statistics and cross tabulation.

## RESULTS

Totally, 595 *Candida* spp. were isolated from the patients. The most sites of the isolated (70%) were mouth and lung (sputum and bronchoalveolar lavage), but *Candida* species were also isolated from the blood, cerebro spinal fluid, sinus biopsy, eyes, pleural and abdominal tap. The most species isolated from the patients was *C. albicans* followed by C. *krusei*, *C. glabrata*, *C. kefyr*, and *C. parapsilosis* (Table 1).

**Table 1:** Distributions of *Candida* species isolates from patients 2005-2009.

Species	No. of isolates	% of isolates
*Candida albicans*	285	48%
*Candida krusei*	96	16.1%
*Candida glabrata*	80	13.5%
*Candida kefyr*	44	7.4%
*Candida parapsilosis*	29	4.8%
*Candida tropicalis*	10	1.7%
Others*	51	8.5%
Total	595	100

* Others include *C. dublinensis* 9, *C.apicola* 8, *C. famata* 4, *C. zeylanoides* 6, *Cryptococcus neoformans* 9, *Trichosporon beigelii*. 8, *Saccharomyces cervicea* 7.

*Candida albicans*, the most species isolated, was sensitive to caspofungin, voriconazole, amphotericine B, ketoconazole, and fluconazole with 98.2%, 94%, 93%, 90.6%, and 89.5%, respectively. Of the 96 *C. krusei* strains, 32 (33.4%) were sensitive to fluconazole, 93 (96.9%) to amphotericine B, 86 (89.6%) to ketoconazole, 77 (80.2%) to voriconazole and 92 (95.8%) to caspofungin. Among the 80 *C. glabrata*, the third isolated species, 32 (40%) were found to be sensitive to fluconazole, 78 (97.5%) to amphotericine B, 68 (85%) to ketoconazole, 72 (90%) to voriconazole and 77 (96.3%) to caspofungin. *Candida kefyr* species had the least sensitivity to fluconazole 24 (54.5%), and high sensitivity (> 85%) to the other antifungals. *Candida Parapsilopsis* species showed high sensitivity rate to all antifungal agents. The lowest sensitivity was seen to itraconazole, *C. albicans* 189 (66.3%), *C. glabrata* 12 (15%), and *C. krusei* 13 (13.5%)**. Table 2 presents the antifungal susceptibility testing of *Candida* isolates to antifungal agents by E-test.

**Table 2:** Distributions of MIC (µg/ml) by E-test method^*a*^.

Species (no. isolates)	Antifungal agent	Range	50%	90%	Number of Resistance (%)
*C.albicans* (285)	Fluconazole	1.00-64.0	4.000	16.00	30.(10.5%)
	Amphotericin	0.032-1.00	0.032	0.500	20 (7%)
	Ketoconazole	0.002-16.00	0.064	4.00	27 (9.4%)
	Itraconazole	0.016-4.00	0.190	2.00	96 (33.7%)
	Voriconazole	0.025- 0.003-16.00	0.064	4.00	17 (6%)
	Caspofungin	1.00	0.025	0.075	5 (1.8%)
*C. krusei* (96)	Fluconazole	4.00-64.00	8.00	128.00	64 (66.6%)
	Amphotericin	0.064-1.00	0.125	0.250	3 (3.1%)
	Ketoconazole	0.380-32.00	1.500	4.00	10 (10.4%)
	Itraconazole	0.500-16.00	0.5.00	4.00	83 (86.5%)
	Voriconazole	0.100-32.00	0.500	2.00	19 (19.8%)
	Caspofungin	0.015-0.5	0.24	0.500	4 (4.2%)
*C. glabrata* (80)	Fluconazole	0.75-256	64.00	128.0	48 (60%)
	Amphotericin	0.013-1	0.190	0.500	2 (2.5%)
	Ketoconazole	0.013-12	1.500	6.00	12 (15%)
	Itraconazole	0.500-16.0	2.00	16.00	68 (85%)
	Voriconazole	0.012-8.00	0.750	3.00	8 (10%)
	Caspofungin	0.03-4.00	0.06	0.12	3 (3.7%)
*C. Kefyr* (44)	Fluconazole	0.038-128.0	1.00	8.000	20 (45.5%)
	Amphotericin	0.016-1.00	0.190	0.750	2 (4.5%)
	Ketoconazole	0.012-0.190	0.032	0.047	1 (2.3%)
	Itraconazole	0.002-1.00	0.047	0.500	5 (11.4%)
	Voriconazole	0.008-16.00	0.023	0.064	1 (2.3%)
	Caspofungin	0.03-1	0.064	0.125	1 (2.3%)
*C. parapsilosis*(29)	Fluconazole	2.00-64.00	1.00	4.00	2 (6.9%)
	Amphotericin	0.023-0.500	0.250	0.500	1 (3.5%)
	Ketoconazole	0.016-0.064	0.023	0.047	1 (3.5%)
	Itraconazole	0.023-0.500	0.125	0.250	1 (3.5%)
	Voriconazole	0.006-0.047	0.016	0.032	0.00
	Caspofungin	0.03-2	0.25	1.00	0.00

Resistance is defined as the following MIC in micrograms per milliliter: Flu ≥ 64; AMB > 1.0; Keto ≥ 4.0; Itra ≥ 1.0; Vori ≥ 4.0; and Caspofungin ≥ 2.0.

Comparing the MIC90 of species, the lowest MIC90 was observed for caspofungin (0.5 µg/ml). The MIC90 for all *Candida* species were 64 µg/ml for fluconazole, 0.75 µg/ml for amphotericin B, 4 µg/ ml for ketoconazole, 4 µg/ml for itraconazole, and 2 µg/ml for voriconazole.

## DISCUSSION

In the present study, the agar-based E-test was used and performed well for the testing of antifungal agents as there are reports about the usefulness of this method and agreement between the E-test and the broth micro-dilution MIC for *Candida* species and different types of antifungal agents (16, 17).

In this study, we isolated 595 strains from various clinical samples with higher rate of C. *albicans* (48%)**, followed by *C. glabrata* (13.5%) and *C. Parapsilosis* (4.8%) (Table 1). The distributions of the species are different in various regions and studies, like 50% *C. albicans*, 24.7% *C. glabrata*, and 1% *C. parapsilosis* in other studies (18, 19). These observations establish the great importance of non *-albicans Candida* as a pathogen in clinical samples. It is important that increase in non-*albicans* species in SC with intensive and long-term use of antifungals leads to higher level of resistance of *Candida* strains to the antifungal drugs (20–22). A remarkable point in our study is that the most commonly isolated species was *C. albicans* in the clinical samples followed by *C. krusei* and *C. glabrata* which can pose a serious threat due to resistance to the routine antifungal agents.

Amphotericin B deoxycholate was the first systemic antifungal agent for the treatment of invasive fungal infections and has been the drug of choice (23, 24), however, due to nephrotoxicity in up to 80% of the patients, use of amphotericin B has been limited (25). Specific breakpoint for amphotericin B has not been proposed because it can positively affect the immune system and stimulates the body defenses against fungal infections (26, 27); therefore, the correlation between in vitro susceptibility pattern and in vivo responses in patients is not predictable. Resistance to amphotericin B as a routine antifungal agent and valid in our hospital for SC, varies in different studies. All *Candida* isolates in Tseng et al. 2005, were susceptible to amphotericin (17) but in our study 7% of *C. albicans* with MIC90, 0.500 µg/ml, 3.1% of *C. krusei* (MIC90 0.250 µg/ml), and 2.5% of *C. glabrata* (MIC90 0.500 µg/ml) were resistant to it.

Development of the triazoles in the 1990s provided alternative options for treating SC. Long-term fluconazole and itraconazole prophylaxis was associated with reduction in susceptibility to these agents. Susceptibility of *C. albicans* to fluconazole in this study was 89.5% (at MIC ≤ 16 µg/mL), comparable with the susceptibility rates reported in other studies (80.9%, 87%, 79% and 87.5%) (17, 28–30). The resistant rate of *C. albicans* to itraconazole in this study was 33.7%, and MIC90% for it was presented ≤ 6 µg/ml. Different rates of resistance to fluconazole and itraconazole were detected in *Candida* strains especially non *albicans* strain (14, 18). From the standpoint of antifungal resistance, *C. glabrata* and *C. krusei* are clearly the *Candida* species with the greatest potential to acquire resistance to fluconazole and other azoles (14, 15). Of the 96 *C. krusei* strains, 83 (86.3%) were resistant to itraconazole, 64 (66.6%) to fluconazole, 19 (19.8%) to voriconazole, 4 (4.2%) to caspofungin, and 3 (3.1%) to amphotericine B. Also, the MIC of *C. glabrata* was higher than that for all other species of *Candida* to triazoles agents (Table 2).

Blood stream infection due to *C. kefyr* is uncommon, but there has been some reports in immunocompromised patients (31) and resistance to antifungals in the literature (4, 20). In the present study, 7.4% of *Candida* isolates were *C. kefyr*, of which 45.5% were resistant to fluconazole but sensitive to the other antifungals.

There were many species which resisted two or three azole antifungal agents and with higher MIC for other azole antifungal agents. This shows that the resistance to one azole can lead to resistance to other azoles, as reported in other studies (14, 15) and thus, is a caution for use of this agent in clinical practice.

Caspofungin is the first echinocandin, approved in 2002 with the mechanism of inhibiting the 1, 3-β d-glucan as an integral part of the fungal cell wall. In the present study, caspofungin was the most active compound inhibiting 90% of *C. albicans* isolates at 0.075 µg/ml, *C. krusei* in 0.5 µg/ml, *C. glabrata* in 0.12 µg/ml; *C. Kefyr* in 0.125 µg/ml; and *C. parapsilosis* in 1.0 µg/ml. Some species such as *C. parapsilosis* and *C. guilliermondii* have a relatively higher echinocandin MIC (32).

There are many view points on the use of new antifungal agents. Such agents are very effective but in many countries, especially in the developing ones, they are very expensive or not available to the respective patients. Therefore, we need to know the antifungal susceptibility rates in each region for the available agents in order to better manage the patients.

The successful treatment of SC depends on the early identification of the species and sensitivity patterns to antifungal agents. The high growing rate of non *albicans Candida* resistant to azole confirms the importance of monitoring changes in the distribution of pathogenic *Candida* species. The sensitivity pattern of *Candida* species as revealed in this study shows that amphotericin B, voriconazole, and caspofungin with the lowest MIC seem to be suitable drugs for empirical therapy and fluconazole and itraconazole are not suitable because of their high MIC and *Candida* species resistance to them.
